# Dissemination of a Pediatric Musculoskeletal POCUS Scoring System via Virtual Education: A Proof-of-Concept Study

**DOI:** 10.24908/pocus.v8i2.16229

**Published:** 2023-11-27

**Authors:** Ysabella Esteban, Jackeline Rodriguez-Smith, Marie Tominna, Amy Cassedy, Arthur B Meyers, Michael Henrickson, Tracy V Ting, Patricia Vega-Fernandez

**Affiliations:** 1 Division of Rheumatology, Nationwide Children's Hospital Columbus, OH USA; 2 Department of Pediatrics, Division of Rheumatology, University of Cincinnati, Cincinnati Children's Hospital Medical Center Cincinnati, OH USA; 3 Beaumont Hospital Royal Oak, MI USA; 4 Department of Pediatrics, Division of Biostatistics and Epidemiology, University of Cincinnati, Cincinnati Children's Hospital Medical Center Cincinnati, OH USA; 5 Department of Radiology, Division of Radiology and Medical Imaging, University of Cincinnati, Cincinnati Children's Hospital Medical Center Cincinnati, OH USA

**Keywords:** musculoskeletal ultrasound, paediatric POCUS, arthritis, point of care ultrasound education

## Abstract

Point of care pediatric musculoskeletal POCUS scanning and scoring protocols for childhood arthritis have emerged in recent years. However, pediatric musculoskeletal POCUS curricula in rheumatology fellowship programs are limited due to availability of trained faculty and resources. This proof-of-concept study investigated the effectiveness of educational methods for a pediatric musculoskeletal POCUS scoring protocol among fellows and physicians of differing subspecialties. Educational methods assessed included recorded videos and virtual review sessions. Effectiveness was assessed by calculating interrater reliability for the musculoskeletal POCUS scoring systems using the intra-class correlation coefficient (ICC). Following training sessions, participants then underwent scoring exercise(s) until the goal of an excellent ICC ≥ 0.75 was reached. Four participants completed two rounds of virtual education, review, and scoring sessions. Excellent interrater reliability was achieved for most views. This proof-of-concept study demonstrated virtual education covering advanced concepts of pediatric musculoskeletal POCUS provides a knowledge base for physicians from different subspecialties and various experience.

## Introduction

In recent years, point of care ultrasound (POCUS) is becoming more prevalent in medical education and in patient care 1. After the onset of the COVID-19 pandemic, there are examples of successful virtual adaptation of POCUS in medical student and radiological resident education 2, 3. From a practical standpoint, the use of musculoskeletal POCUS is becoming more widespread and ubiquitous within medicine, specifically within the field of rheumatology. In addition, a previous study demonstrated successful teaching of sonographic findings of inflammatory arthritis to medical students, based on utilization of a multiple-choice exam, practical skills assessment, and overall score determined by the educators 4.

Advancing a POCUS skillset relies mostly on self-directed education and/or enrollment in specific curricula 5, 6, 7, 8. In recent years, musculoskeletal POCUS training has been integrated into many adult rheumatology fellowship programs, with over 100 programs in the United States providing education on this imaging modality. However, the availability of standardized curricula varies between fellowship programs 5. In an effort to support musculoskeletal POCUS training, the American College of Rheumatology (ACR) introduced a musculoskeletal POCUS educational curriculum for adult patients, with related appendices for pediatric patients 6. In order to quantify the availability of pediatric musculoskeletal POCUS curricula, one recent cross-sectional study showed that 20 of the 36 ACGME-accredited pediatric rheumatology fellowship programs in the United States and Canada offer some level of musculoskeletal POCUS training 8. However, the dissemination of pediatric musculoskeletal POCUS curricula can be limited due to the availability of trained faculty, constraints on time and resources to perform these studies, and decreased awareness of available curricula 8. 

Within the context of clinical applications of pediatric musculoskeletal POCUS, there exists definitions for pediatric sonographic findings of healthy joints as well as sonographic findings of inflammatory arthritis 9, 10, 11. Recent studies within the field of pediatric rheumatology and musculoskeletal POCUS have focused on the development of pediatric-specific scanning protocols for the assessment of synovitis in juvenile idiopathic arthritis(JIA) and corresponding scoring systems, including those proposed by the Outcome Measures in Rheumatology (OMERACT) and Childhood Arthritis and Rheumatology Research Alliance (CARRA) ultrasound work groups 12, 13, 14, 15, 16, 17. However, the establishment of curricula for advanced concepts including consensus-based scoring systems for pediatric synovitis within the context of rheumatic disease has not been well explored. 

This proof-of-concept study aims to explore this knowledge gap, and to provide further insight into educational opportunities within the field. Here, we provided an initial assessment of 1) the educational methods used to teach and 2) the reproducibility of a pediatric-specific musculoskeletal POCUS scoring system among fellows-in-training and a pediatric radiology attending. 

## Materials and Methods

**Figure 1:**
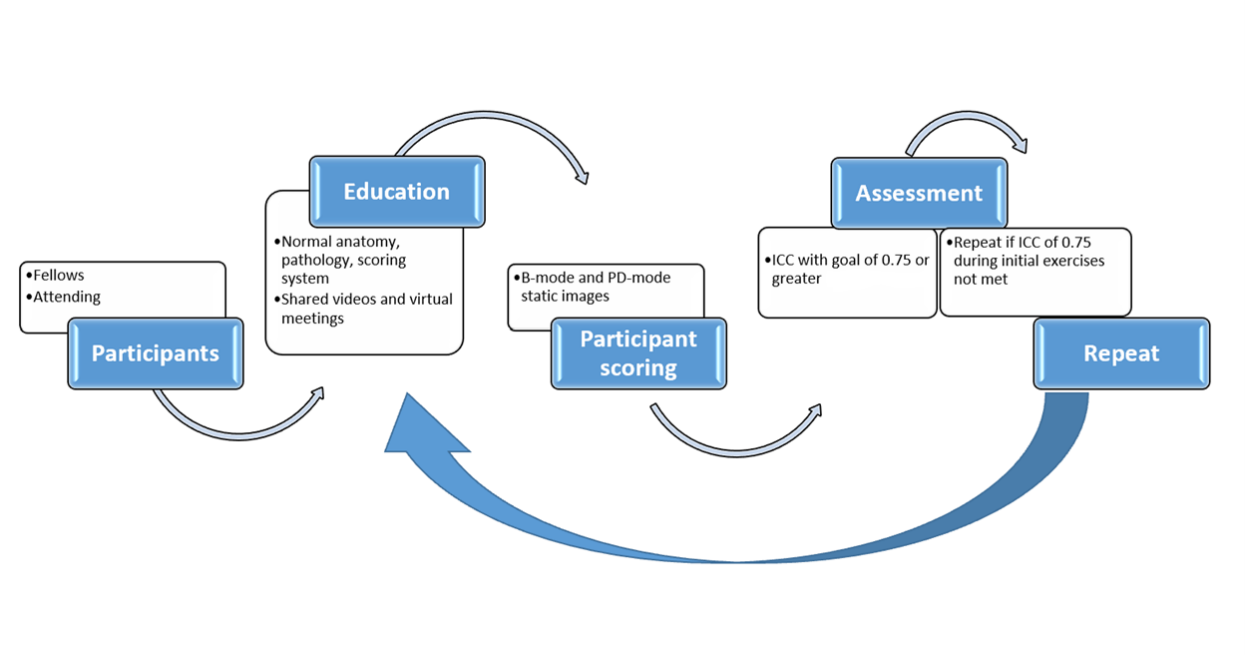
Methods. Methodology used for education and scoring exercises. Participants included 3 fellows in training from pediatric rheumatology (2) and pediatric radiology (1), as well as 1 attending from musculoskeletal radiology. Sessions were led by a trained pediatric Musculoskeletal Ultrasound Certification in Rheumatology (RhMSUS) certified sonographer of >10 years. Education materials covered normal sonographic anatomy, pathologic findings related to arthritis, as well as a semiquantitative scoring system.^13^, ^15^ Scoring exercises were comprised of still images using B- and PD-mode, representative of normal and pathologic findings. Assessment used intra-class correlation coefficient (ICC) with goal of 0.75 or greater for the lower confidence interval (CI) for each joint.^18^, ^19^ Education and scoring were repeated if this goal was not achieved per joint and view.

This was a proof-of-concept study, and participants included those with various degrees of training and experience in musculoskeletal POCUS (from 1 year to >10 years), including three fellows (two pediatric rheumatology fellows, one pediatric radiology fellow) and one radiology attending with expertise in musculoskeletal imaging and 10 years of post-training experience. The education provided for the group included two modalities: 1) recorded educational videos and 2) follow-up review sessions via virtual meet space. These videos and review sessions were guided by an expert pediatric rheumatologist and ultrasonographer (PVF, 10 years of musculoskeletal POCUS experience). The methods are outlined in Figure 1. 

The recorded educational videos were led by the musculoskeletal POCUS expert (PVF) and facilitator (YE), who helped guide discussion points during the recording and review sessions. The videos reviewed the sonographic anatomy as well as pathologic findings for B-mode and PD-mode of the elbow, wrist, finger, knee, and ankle joints. During these videos, the scoring system for each joint was also taught, using static images obtained from patients 2-17 years of age. Briefly, the definitions of pathology used by the scoring systems followed the recommendations proposed by the OMERACT Pediatric Ultrasound Group 7. The scoring systems used a semi-quantitative scale (0-normal to 3-severe) to categorize the findings of synovitis in B-mode and power Doppler (PD) images of the aforementioned joints (Table 1) 13, 15, 16, 17. 

**Table 1:** Anatomical Views for Elbow, Wrist, Finger, Knee, and Ankle Joints 13, 15, 16, 17

**Joint**	**Anatomical Views (B-mode and PD-mode)**
Elbow	Anterior humeroulnar joint recess Anterior humeroradial joint recess Posterior humeroulnar joint recess
Wrist	Radiocarpal and midcarpal joint recess – midline Radiocarpal and midcarpal joint recess – ulnar Distal radioulnar joint recess Extensor tendons
Finger	MCP dorsal joint recess in longitudinal MCP volar joint recess in longitudinal PIP volar joint recess in longitudinal PIP dorsal joint recess in longitudinal
Knee	Suprapatellar joint recess Medial parapatellar joint recess Lateral parapatellar joint recess
Ankle	Anterior tibiotalar joint recess Talonavicular joint recess Anterior subtalar joint recess (from medial aspect) Posterior subtalar joint recess (from lateral aspect) Anterior, medial, and lateral tendons
Anatomical views for the scoring protocol as determined by previous studies. 13, 15, 16, 17	

The duration of each video ranged from 24 to 46 minutes in length. In addition, electronic handouts were provided to participants, which detailed the anatomy, pathology, and scoring system of each joint. Participants were encouraged to complete the videos prior to the virtual review sessions. 

The two review sessions then took place via a virtual meet space, led by YE and PVF, which lasted approximately 1.5 – 2 hours total. During this review, participants asked questions regarding the anatomy, pathology, and scoring system. Then, participants were able to practice the scoring system, and real-time feedback and discussion were provided based on the score each participant chose. Following the review sessions, participants had more opportunities to individually review the material on their own time, and one-on-one question and answer sessions regarding the scoring system via phone or email were provided as needed. 

All participants then underwent a scoring exercise which was conducted electronically, using B- and PD-mode static images from a previously existing pediatric musculoskeletal POCUS image bank obtained from children 2 – 17 years and representing the spectrum of pathology described by the scoring systems for each joint. The images were selected based on the quality of the image, specifically considering clear delineation of bony/cartilage landmarks and the least amount of anisotropy or artifact. The images selected for the review sessions were different than those selected for the scoring exercises. No identifiable patient information was presented with the images save for the age of the subject. The images were scored independently by each participant; statistical analysis followed each exercise. Based on the results from the first scoring exercise, participants underwent a second review session with emphasis on the areas that did not reach excellent reliability as defined by an intra-class correlation coefficient (ICC) ≤0.75 18, 19. During this live virtual review session, additional sample images for scoring practice were provided, with participants providing scores followed by discussion as to why specific scores were chosen. Then, a second scoring exercise was performed which focused on those particular areas utilizing B- and PD-mode static images. 

The interrater reliability was estimated using the two-way single score ICC, along with the 95% confidence intervals (CI). Excellent ICC was defined to be between 0.75 – 1.00, good 0.60 – 0.74, fair 0.40 – 0.59, and poor <0.40 18, 19. ICC estimates and their 95% confidence intervals were calculated using SAS v9.4©, Cary, NC. 

This study was submitted to the authors’ Institutional Review Board (Cincinnati Children’s Hospital Medical Center) and received exemption status: IRB# 2020-0700. 

## Results

A total of four raters participated in this proof-of-concept study. A total of two rounds of educational and scoring sessions were completed. The results from the first educational and scoring exercise are listed in Table 2. For this scoring exercise, a total number of 588 images representing both normal sonographic anatomy as well as varying degrees of pathology related to the scoring system (including 352 B-mode and 236 PD-mode still images) were scored. Based on these results, excellent interrater reliability (ICC ≥ 0.75) was achieved for most of the B-mode and PD mode views of the elbow, wrist, finger, knee and ankle as delineated in Table 2. 

**Table 2:** Interrater Reliability of Pediatric-Specific musculoskeletal POCUS Scoring System– Exercise 1 and 2.

**Joint View**		**Exercise 1**		**Exercise 2**	
		**B-mode** **ICC (95% CI)**	**Power Doppler-Mode** **ICC (95% CI)**	**B-mode** **ICC (95% CI)**	**Power Doppler-Mode** **ICC (95% CI)**
**Elbow**	Anterior humeroulnar and humeroradial joint recesses	0.93 (0.89 – 0.95)	0.88 (0.77 - 0.94)	^‡^	^‡^
	Posterior humeroulnar joint recess	0.93 (0.89 - 0.95)	0.77 (0.61 - 0.87)	^‡^	^‡^
**Wrist**	Radiocarpal and midcarpal joint -- midline	0.86 (0.80 - 0.90)	0.96 (0.94 - 0.97)	^‡^	^‡^
	Radiocarpal and midcarpal joint -- ulnar	0.80 (0.65 - 0.89)	0.90 (0.71 - 0.97)	^‡^	^‡^
	Distal radioulnar joint recess	0.87 (0.78-0.92)	0.93 (0.94 – 0.97)	^‡^	^‡^
	Tendons -- extensor	0.74* (0.58 - 0.84)	0.66* (0.35 - 0.84)	0.5* (0.19 – 0.72)	0.52* (0.19 – 0.74)
**Finger**	MCP dorsal joint recess in longitudinal	0.94 (0.90 - 0.96)	0.98 (0.97 - 0.99)	^‡^	^‡^
	MCP volar joint recess in longitudinal	0.82 (0.73 - 0.88)	0.89 (0.83 - 0.93)	^‡^	0.95 (0.93-0.97)
	PIP volar joint recess in longitudinal	0.91 (0.87 - 0.94)	0.63* (0.48 - 0.74)		
	PIP dorsal joint recess in longitudinal	0.94 (0.88-0.97)	0.84 (0.70 - 0.92)	^‡^	^‡^
**Knee**	Suprapatellar joint recess	0.93 (0.89 - 0.95)	0.88 (0.82 - 0.92)	^‡^	^‡^
	Medial parapatellar joint recess	0.92 (0.88 - 0.95)	0.90 (0.85 – 0.93)	^‡^	^‡^
	Lateral parapatellar joint recess	0.94 (0.91 – 0.96)	0.90 (0.84 – 0.93)	^‡^	^‡^
**Ankle **	Anterior tibiotalar joint recess	0.92 (0.88 – 0.95)	0.87 (0.75 – 0.93)	^‡^	^‡^
	Talonavicular joint recess	0.66* (0.41 - 0.82)	0.91 (0.8 - 0.96)	0.84 (0.71-0.91)	^‡^
	Anterior subtalar joint recess (medial aspect)	0.77 (0.63 - 0.86)	0.83 (0.59 - 0.94)	^‡^	^‡^
	Posterior subtalar joint recess (lateral aspect)	0.73* (0.57 - 0.84)	^†^	0.90 (0.85-0.96)	0.91 (0.83-0.95)
	Tendons—Medial, lateral and anterior	0.53* (0.26 - 0.73)	0.55* (0.1 - 0.81)	0.82 (0.73-0.88)	0.95 (0.91-0.97)
*Desired reliability of ≥ 0.75 not obtained. ^†^Insufficient data to assess. ^‡^Not applicable Interrater reliability was estimated using ICC. Excellent ICC was defined to be between 0.75-1.00, good 0.60-0.74, fair 0.40 – 0.59, and poor < 0.40. [18, 19]					

For the remaining views in which excellent reliability was not reached, a second round of education and scoring exercises was performed, with the results listed in Table 2. For this scoring exercise, a total number of 234 B-mode and PD-mode still images were scored. Desired excellent interrater reliability was obtained for the remaining B-mode and PD-mode views with the exception of those delineating the tendons of the wrists. For this area, a fair reliability was obtained. 

Feedback regarding the educational sessions was obtained in real-time from participants. Positive feedback included the flexibility of the curriculum given the video format with electronic handouts, as well as the structure of educational sessions. Suggestions for improvement included incorporating more examples of image scoring for both B-mode and PD-mode views during the interactive virtual review sessions. 

## Discussion

This proof-of-concept study explored the ability of pediatric physicians with varying degrees of expertise to learn and demonstrate a novel pediatric musculoskeletal POCUS scoring system. From an educational standpoint, the information provided to participants built upon basic musculoskeletal anatomy, and participants were able to gain or fine tune knowledge regarding joint pathology as visualized on B- and PD-modes. Knowledge of the scoring protocol was also imparted. The format of the educational sessions, which included video lectures encompassing the related sonographic anatomy, pathology, and scoring system of each joint, allowed participants to learn independently. In addition, during the virtual, interactive review sessions the participants were able to practice scoring, concept review, and discussion of the scoring systems in real-time. Immediate, informal feedback at the end of each session allowed incorporation of suggestions into subsequent educational sessions. 

Use of musculoskeletal POCUS facilitates real-time diagnosis, intervention, and monitoring by the clinician ultrasonographer 20, 21. In addition, the evolution of technology has provided the medical community with multiple portable options, which has allowed pediatric providers increased access to this mode of imaging 21. In terms of musculoskeletal POCUS, there are multiple routes of education including rheumatology fellowship training, mentored training that is either structured or unstructured, or self-directed and non-mentored training 20. For pediatric musculoskeletal POCUS, basic educational opportunities include the training program offered by the Ultrasound School of North American Rheumatologists (USSONAR), workshops and courses previously offered by the American College of Rheumatology (ACR) or Childhood Arthritis Rheumatology Research Alliance (CARRA) in the pre-COVID era, as well as self-directed online resources such as Ped-MUS 6, 22, 23. Twenty pediatric rheumatology fellowship programs also offer musculoskeletal POCUS training 8. 

To our knowledge, this is the first effort to investigate a virtual educational format to train pediatricians in the assessment of musculoskeletal POCUS studies as they pertain to JIA using advanced concepts**. **While it is possible that the size and flexibility of this participant group facilitated this investigation, it is feasible that these educational sessions can be replicated among larger participant groups in the future. The pandemic has accelerated the era of virtual meetings, which some have found to be beneficial in radiological education 3, and our demonstration of teachability and reproducibility of this scoring system via a virtual meet space is likewise encouraging. The era of COVID-19 proved to be a new obstacle in live in-person musculoskeletal POCUS training but also provided the impetus for developing virtual, remote learning options, which will likely continue to be an integral part of this training 24. This also highlights the potential to provide virtual education for clinicians in underserved areas, including global outreach programs. 

The limitations of this study included the reduced number of participants involved. A lower ICC may reflect not only the degree of interrater agreement, but also a smaller number of raters or the diverse experience of the raters. In our particular study, we had four participants, which could have contributed to this finding. Competency assessment of the proposed online curriculum was not pursued mainly given the size of the team evaluated. In addition, solicited feedback was not anonymous given the size of the group. 

The one area that only attained fair interrater reliability after the second round of training in this study was B-mode and PD-mode views for the tendons of the wrists. One possible explanation for this is that scoring involved static images, as opposed to cine clips which can better distinguish hypoechoic muscle surrounding a tendon at the myotendinous junction from fluid. In addition, the presence of the retinaculum adjacent to the dorsal wrist tendons can exhibit a hypoechoic appearance, which without dynamic study to assess for anisotropy or compressibility, could potentially affect interpretation by mimicking tenosynovitis 25, 12, 13, 14, 15, 21. 

Finally, as this was a proof-of-concept study, we did not perform longitudinal follow-up of participant knowledge recall and therefore cannot comment on retention. Future qualitative studies should investigate areas of optimization in education of this pediatric musculoskeletal scoring system among varying levels of expertise, including pre- and post-curriculum knowledge assessment, long-term follow-up, and where applicable, impact on clinical practice. 

## Conclusion

Our study showed that virtual educational exercises covering normal musculoskeletal POCUS anatomy and pathologic variations related to pediatric arthritis can provide a knowledge base to physicians from different subspecialties at varying points in their training and careers. Further qualitative studies should be performed to assess areas of optimization in education of pediatric musculoskeletal POCUS and pediatric-specific musculoskeletal POCUS scoring systems. Finally, our study demonstrated that a virtual platform for pediatric musculoskeletal POCUS curricula is a feasible option, and could be implemented for pediatric rheumatology fellowship programs that do not have ready access to trained faculty or resources. 

## Disclosures

Dr. Vega-Fernandez’s work was supported by the Center for Clinical & Translational Science & Training (CCTST) at the University of Cincinnati funded by the National Institutes of Health (NIH) Clinical and Translational Science Award (CTSA) program, grant 2UL1TR001425-05A1 and KL2 (2KL2TR001426-05A). The content is solely the responsibility of the authors and does not necessarily represent the official views of the NIH.

## References

[R213253129459577] Tarique U, Tang B, Singh M, Kulasegaram K M, Ailon J (2017). Ultrasound Curricula in Undergraduate Medical Education: A Scoping Review. J Ultrasound Med.

[R213253129459585] Zavitz J, Sarwal A, Schoeneck J, Glass C, Hays B, Shen E, Bryant C, Gupta K (2021). Virtual multispecialty point-of-care ultrasound rotation for fourth-year medical students during COVID-19: Innovative teaching techniques improve ultrasound knowledge and image interpretation. AEM Educ Train.

[R213253129459595] Larocque N, Shenoy-Bhangle A, Brook A, Eisenberg R, Chang Y M, Mehta P (2021). Resident Experiences With Virtual Radiology Learning During the COVID-19 Pandemic. Acad Radiol.

[R213253129459594] Wright S A, Bell A L (2008). Enhancement of undergraduate rheumatology teaching through the use of musculoskeletal ultrasound. Rheumatology (Oxford).

[R213253129459589] Torralba K D, Cannella A C, Kissin E Y, Bolster M B, Salto L M, Higgs J, Samuels J, Nishio M J, Kaeley G S, Evangelisto A, Marco P De, Kohler M J (2020). Musculoskeletal Ultrasound Instruction in Adult Rheumatology Fellowship Programs. Arthritis Care Res (Hoboken).

[R213253129459587] Cannella A C, Kissin E Y, Torralba K D, Lin C, Higgs J B, Bolster M (2019). Rheumatologic Ultrasound (RhUS) Curriculum Supplement. American College of Rheumatology.

[R213253129459586] Widener B B, Cannella A, Martirossian L, Kissin E Y (2020). Modern Landscapes and Strategies for Learning Ultrasound in Rheumatology. Rheum Dis Clin North Am.

[R213253129459582] Lin C, Oberle E, Curran M, Roth J, Vega-Fernandez P, Deranieri D, Ting T, Benham H, Salto L, Torralba K (2021). Musculoskeletal Ultrasound Instruction in US and Canadian Pediatric Rheumatology Fellowship Programs. Poster presented at: ACR Convergence 2021.

[R213253129459593] Roth J, Ravagnani V, Backhaus M, Balint P, Bruns A, Bruyn G A, Collado P, Cruz L De La, Guillaume-Czitrom S, Herlin T, Hernandez C, Iagnocco A, Jousse-Joulin S, Lanni S, Lilleby V , Malattia C, Magni-Manzoni S, Modesto C, Rodriguez A, Nieto J C, Ohrndorf S, L Rossi-Semerano, Selvaag A M , Swen N, Ting T V , Tzaribachev N, Vega-Fernandez P, Vojinovic J, Windschall D, D'Agostino M A, Naredo E, Omeract Ultrasound Group (2017). Preliminary Definitions for the Sonographic Features of Synovitis in Children. Arthritis Care Res.

[R213253129459588] Roth J, Jousse-Joulin S, Magni-Manzoni S, Rodriguez A, Tzaribachev N, Iagnocco A, Naredo E, D'Agostino M A, Collado P, Outcome Measures in Rheumatology Ultrasound Group (2015). Outcome Measures in Rheumatology Ultrasound Group. Definitions for the sonographic features of joints in healthy children. Arthritis Care Res.

[R213253129459574] Collado P, Windschall D, Vojinovic J, Magni-Manzoni S, Balint P, Bruyn Gaw, Hernandez-Diaz C, Nieto J C, Ravagnani V, Tzaribachev N, Iagnocco A, D'Agostino M A, Naredo E (2018). OMERACT ultrasound subtask force on pediatric. Amendment of the OMERACT ultrasound definitions of joints' features in healthy children when using the DOPPLER technique. Pediatr Rheumatol Online J.

[R213253129459575] Sande N K, Bøyesen P, Aga A B, Hammer H B, Flatø B, Roth J, Lilleby V (2021). Development and reliability of a novel ultrasonographic joint-specific scoring system for synovitis with reference atlas for patients with juvenile idiopathic arthritis.. RMD Open.

[R213253129459590] Ting T V, Vega-Fernandez P, Oberle E J, Ranieri D De, Bukulmez H, Lin C, Moser D, Barrowman N J, Zhao Y, Benham H M, Tasan L, Thatayatikom A, Roth J (2019). Childhood Arthritis and Rheumatology Research Alliance Juvenile Idiopathic Arthritis Ultrasound Workgroup. Novel Ultrasound Image Acquisition Protocol and Scoring System for the Pediatric Knee. Arthritis Care Res (Hoboken).

[R213253129459592] Rossi-Semerano L, Breton S, Semerano L, Boubaya M, Ohanyan H, Bossert M, Boiu S, Chatelus E, Durand G, Jean S, Goumy L, Mathiot A, Mouterde G, Nugues F, Hennia A Ould, Rey B, Scheven A Von, Sparsa L, Devauchelle-Pensec V, Jousse-Joulin S (2021). Application of the OMERACT synovitis ultrasound scoring system in juvenile idiopathic arthritis: a multicenter reliability exercise. Rheumatology.

[R213253129459597] Vega-Fernandez P, Ting T V, Oberle E J, Mccracken C, Figueroa J, Altaye M, Cassedy A, Kaeley G S, Roth J, Carra Musculoskeletal Ultrasound Workgroup (2021). The MUSICAL pediatric ultrasound examination - a comprehensive, reliable, time efficient assessment of synovitis.. Arthritis Care Res.

[R213253129459580] Vega-Fernandez P, Ranieri D De, Oberle E, Clark M, Bukulmez H, Lin C, Shenoi S, Thatayatikom A, Woolnough L, Benham E, Brunner E, Henrickson M, Pratt L R, Proulx-Gauthier J P, Janow G, Cassedy A, Ting T V, Roth J, Carra Jia Ultrasound Workgroup (2022). Comprehensive and reliable sonographic assessment and scoring system for inflammatory lesions of the pediatric ankle. Rheumatology.

[R213253129459579] Vega-Fernandez P, Esteban Y, Oberle E, Proulx-Gauthier J P, Clark M, Shenoi S, Thatayatikom A, Benham H, Brunner E J, Woolnough L, Henrickson M, Pratt L R, Ranieri D De, Hoffmann S, Janow G, Bukulmez H, Altaye M, Cassedy A, Ting T V, Roth J, Carra Jia Ultrasound Workgroup (2022). Reliability of the Pediatric Specific Musculoskeletal Ultrasound Scoring Systems for the Elbow, Wrist, and Finger Joints. J Rheumatol.

[R213253129459583] Cicchetti D V (1994). Guidelines, criteria, and rules of thumb for evaluating normed and standardized assessment instruments in psychology. Psychological Assessment.

[R213253129459584] Hallgren K A (2012). Computing Inter-Rater Reliability for Observational Data: An Overview and Tutorial. Tutor Quant Methods Psychol.

[R213253129459591] Widener B B, Cannella A, Martirossian L, Kissin E Y (2020). Modern Landscapes and Strategies for Learning Ultrasound in Rheumatology. Rheum Dis Clin North Am.

[R213253129459578] Conlon T W, Nishisaki A, Singh Y, Bhombal S, Luca D De, Kessler D O, Su E R, Chen A E, Fraga M V (2019). Moving Beyond the Stethoscope: Diagnostic Point-of-Care Ultrasound in Pediatric Practice. Pediatrics.

[R213253129459576] Alliance CARRA - Childhood Arthritis and Rheumatology Research (2019). Childhood Arthritis and Rheumatology Research Alliance 2019 Annual Meeting. https://carragroup.org/meetings-events/past-carra-annual-scientific-meetings/2019-annual-meeting.

[R213253129459581] Roth J, Malattia C, Windschall D Ped-MUS.

[R213253129459573] Matteo A Di, Mankia K, Filippucci E, Grassi W, Rowbotham E, Wakefield R J (2021). Facing the challenges of running a rheumatology-based ultrasound service in the COVID-19 era.. Rheumatology.

[R213253129459596] Chiavaras M M, Jacobson J A, Yablon C M, Brigido M K, Girish G (2014). Pitfalls in wrist and hand ultrasound. AJR Am J Roentgenol.

